# Periodic Precipitation in a Confined Liquid Layer

**DOI:** 10.1021/acs.jpclett.4c00832

**Published:** 2024-04-30

**Authors:** Masaki Itatani, Yuhei Onishi, Nobuhiko J. Suematsu, István Lagzi

**Affiliations:** † Department of Physics, Institute of Physics, Budapest University of Technology and Economics, Műegyetem rkp. 3, Budapest H-1111, Hungary; ‡ Graduate School of Advanced Mathematical Sciences, Meiji University, 4-21-1 Nakano, Tokyo 164-8525, Japan; § Meiji Institute for Advanced Study of Mathematical Sciences (MIMS), Meiji University, 4-21-1 Nakano, Tokyo 164-8525, Japan; ∥ HU-REN-BME Condensed Matter Physics Research Group, Budapest University of Technology and Economics, Műegyetem rkp. 3, Budapest H-1111, Hungary

## Abstract

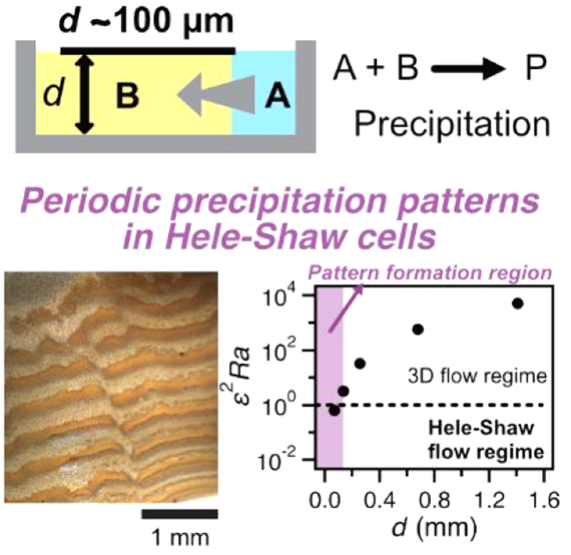

Pattern formation is a ubiquitous phenomenon in animate and inanimate systems generated by mass transport and reaction of chemical species. The Liesegang phenomenon is a self-organized periodic precipitation pattern always studied in porous media such as hydrogels and aerogels for over a century. The primary consideration of applying the porous media is to prevent the disintegration of the precipitation structures due to the sedimentation of the precipitate and induced fluid flow. Here, we show that the periodic precipitation patterns can be engineered using a Hele–Shaw cell in a confined liquid phase, restricting hydrodynamic instability. The patterns generated in several precipitation reaction systems exhibit spatiotemporal properties consistent with patterns obtained in solid hydrogels. Furthermore, analysis considering the Rayleigh–Darcy number emphasizes the crucial role of fluidity in generating periodic precipitation structures in a thin liquid film. This exploration promises breakthroughs at the intersection of fundamental understanding and practical applications.

Reaction–diffusion systems combine diffusion (as a mass transport) and chemical reaction networks, resulting in self-organized static spatial or spatiotemporal patterns.^1−8^ Such pattern formation has been commonly observed in animate^9,10^ and inanimate^11−13^ systems. One of the well-known classes of chemical self-organization in reaction–diffusion systems is the periodic precipitation or Liesegang phenomenon, which creates a periodic precipitation pattern known as the Liesegang pattern (LP).^14,15^ Depending on the geometry of the reaction front, three different pattern structures can be obtained: bands,^16,17^ concentric rings,^18,19^ and onion-like layered shells in 3D.^20^ The LP formation takes place in a porous medium, typically a solid hydrogel, where a homogeneously distributed electrolyte (B, inner electrolyte) is in contact with another medium containing another electrolyte (A, outer electrolyte) (Figure 1a).^15^ This allows A to diffuse into the gel and react with B, forming a periodic array of precipitate. The obtained periodic structure can be characterized by the following equation:
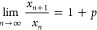
1where *n* is the band number, *x*_*n*_ and *x*_*n*+1_ are the distance of the *n*th and (*n* + 1)th bands measured from the interface between media containing the outer and inner electrolytes, and *p* is the spacing coefficient. This empirical rule is known as the spacing law. This type of structure is often observed as geological patterns,^21,22^ and some researchers have been working to explain the formation mechanism of such geoscientific patterns by the Liesegang phenomenon.^23,24^ Therefore, exploring the LP formation mechanism from a physicochemical standpoint is an essential scientific proposition.

**Figure 1:**
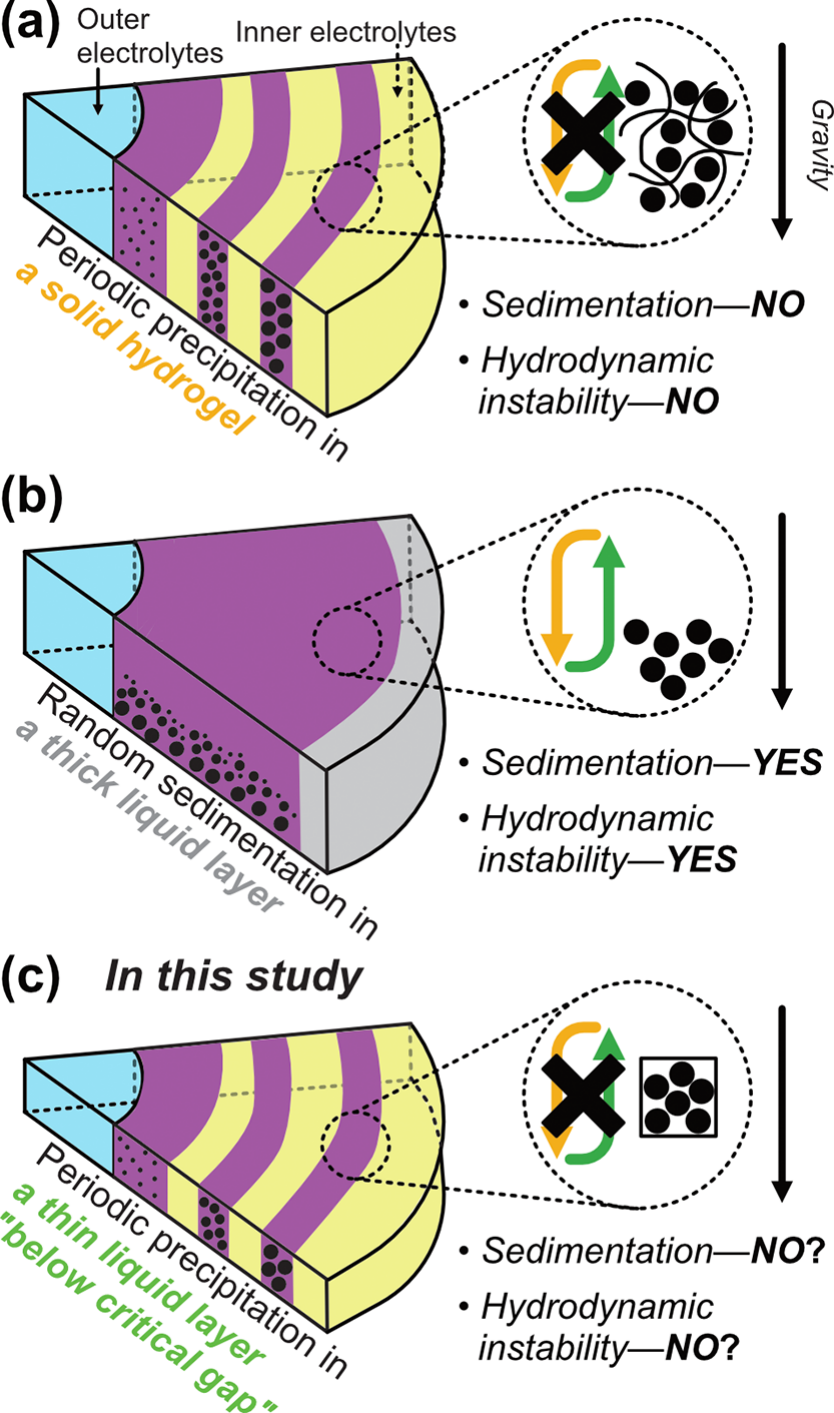
Role of reaction media for precipitation in reaction–diffusion systems. The sketch of the experimental system of (a) classical setup using a solid hydrogel for Liesegang phenomena, (b) an exceptional experimental setup using a thick liquid layer (vertical gap ∼ cm) instead of a gel, where 3D fluid effects such as the buoyancy-driven flow can disturb processes for pattern formation, and (c) a focused setup in this study using a liquid layer confined in Hele–Shaw cells having a smaller gap than a critical value that ensures 2D Hele–Shaw flow, where the 3D effects are negligible.

The Liesegang phenomenon has also attracted attention for its potential applications in material science. Recently, some studies reported the synthesis of colloidal particles with different morphologies, such as gold nanoparticles^25^ and metal–organic frameworks (MOFs),^26−29^ some of which have shown novel shapes which cannot be engineered in batch experiments. However, this potential use of LPs from laboratory scale to industrial applications has been hindered by the complicated extraction processes of particles from the gel using toxic solvents (e.g., *N*,*N*-dimethylformamide).^27,29^ Therefore, finding a way to form LPs in gel-free media, especially a liquid phase, can be beneficial since it makes extraction processes easy, simple, and environmentally friendly.

Since the discovery of the LP,^14^ over the past 120 years, all investigations and studies have always used porous media such as solid hydrogels (e.g., gelatin,^19,30^ agarose,^16,31^ agar–agar,^32,33^ poly(vinyl alcohol),^34,35^ and polyacrylamide^36,37^) and aerogels.^38^ This is because of some practical reasons, in which it is easy to experimentally separate two phases of outer and inner electrolytes or one in which geometries, shapes, and dimensions of the medium can be on demand.^20,31,39−42^ The main argument about the role of the gel is that it prevents sedimentation of colloids and hydrodynamic instability caused by the density difference because the formed precipitate is captured by a polymer network at a position where the reaction occurs (Figure 1a). In addition, recent studies have shown that the morphology of periodic patterns can be controlled using a gel containing different types of polymers^43,44^ or by tuning the gel concentration.^16,31^ The accepted explanation for this effect is that a mesoscopic porous hydrogel structure acts as a heterogeneous nucleation site facilitating the nucleation. However, the role of gel is usually not considered in mathematical models except in the above studies, even though numerical simulation is used in many other studies as a powerful tool to investigate the mechanism.^30,45−48^ Therefore, the essentiality of gel media for LP formation remains to be determined.

On the other hand, from a fluid physics perspective, the pattern must be disturbed, and particles are sedimented randomly in space if a thick liquid layer of more than sub-millimeters is used as the reaction media instead of the gel because of high fluidity and occurrence of density difference-driven convection, namely the lack of the above prevention effect (Figure 1b). However, the liquid layer confined in a narrow space below “a critical gap” should have the same role as the gel since such a narrow space contributes to an increase in the fluid viscosity,^49,50^ which is possible to restrict the sedimentation and hydrodynamic instability, potentially resulting in periodic precipitation in gel-free media (Figure 1c). It should be noted that in the work of Nadir Kaplan et al., the growth of coral-like structures produced three zones in a microfluidic chamber with a height of 150–200 μm.^51^

As a proof of concept to demonstrate whether LPs can be formed in a thin liquid layer or not, in this study, a Hele–Shaw (HS) cell having 70 μm of vertical gap distance (*d*) is applied as a 2D Liesegang experimental setup with some previously well-studied precipitation systems, including CuCrO_4_,^16,17,52^ Ag_2_Cr_2_O_7_,^30,44,53^ and zeolitic imidazolate framework-8 (ZIF-8)^26,27,29^ (for details of the experimental setup, see the Supporting Information and Figure S1). HS cells have already been successfully applied in investigating and generating various precipitation structures utilizing fluid flow^54,55^ and the precipitation reaction.^56−60^ We found that periodic precipitation patterns of CuCrO_4_ obtained fulfilled typical features of LPs such as the spacing law (eq 1), Matalon–Packter law,^45,61^ time law,^62,63^ and width law.^64,65^ To extend our concept, the precipitation of Ag_2_Cr_2_O_7_ and ZIF-8 was also investigated. Then, the critical gap to give periodic structure was finally investigated using a different HS cell experimentally and discussed based on the Rayleigh–Darcy number considering the effects of the geometry of HS cells, which estimates the relative strength of convection to diffusion.^66−69^ This highlights that the fluidity of electrolyte media has a crucial role in the Liesegang phenomenon regardless of the gel presence.

In the initial series of experimental investigations, we aimed to check the existence of the Liesegang phenomenon in a liquid layer confined to a HS cell. The experiments used a commercially available HS cell with a vertical gap distance (*d*) of 70 μm. The setup involved placing a CuCl_2_ aqueous solution (outer electrolyte) outside the HS cell window, in contact with a K_2_CrO_4_ aqueous solution (inner electrolyte) confined to the HS cell at the edge of the cell window (Figure S1). In all cases, the initial concentration of CuCl_2_ ([CuCl_2_]_0_) was deliberately set higher than the initial concentration of K_2_CrO_4_ ([K_2_CrO_4_]_0_), following the typical experimental setup for investigating Liesegang phenomena.^15^ This configuration facilitated the preferential diffusion of Cu^2+^ into the liquid phase containing CrO_4_^2–^, leading to a reaction between Cu^2+^ and CrO_4_^2–^ that produced CuCrO_4_ precipitates (Cu^2+^(aq) + CrO_4_^2–^(aq) → CuCrO_4_(s)), primarily occurring in the confined layer of the HS cell. Using this setup, periodic precipitation patterns were observed with varying [CuCl_2_]_0_ (ranging from 0.5 to 1.5 M) against the fixed [K_2_CrO_4_]_0_ at 0.2 M (Figure 2a). The pattern consisted of a sequence of two repeated zones: an orangish banding structure with a high density of CuCrO_4_ precipitates and an interband structure with a low density of the precipitated particles.^70^ The region where there was no precipitate near the edge of the HS cell was formed because of the invasion of CuCl_2_ solution into the cell due to the capillary effect when the two phases were initially in contact. However, this empty region did not expand during the reaction. Additionally, the separation between regions with and without precipitates remained clear. As one of the observed trends, the position of the reaction front at the given time increased with an increase in [CuCl_2_]_0_ because the diffusion flux of Cu^2+^ increased, which was often observed in some previous studies.^52,61^ Unlike conventional concentration conditions of the inner electrolyte used for CuCrO_4_ LPs with agarose gel media,^16,36^ our setup required the higher concentration ([K_2_CrO_4_]_0_ = 0.2 M) rather than the typical value (0.01 M). As discussed above, gels can facilitate nucleation. However, our experimental configuration diverged from this convention by not using gels. Instead, we relied on an elevated concentration of reactants to augment the nucleation rate. Furthermore, in contrast to the previous studies, some particles appeared in interband regions. Ideally, most of the inner electrolytes near the precipitation band should be consumed to generate particles in the bands. Therefore, it makes the nucleation and growth less significant in the interband region, resulting in a smaller number of particles in the case of lower electrolyte concentration used. Nevertheless, in our study, a greater concentration of CrO_4_^2–^ (sufficient to drive the reaction) persisted in the inter-region despite the ongoing process described above due to the high [CrO_4_^2–^]_0_. As supporting evidence, the density of precipitates in the interband region was lower than within the band. It is also noted that dislocation in the periodic structure, such as branching and position displacement of bands, was observed in most of our experiments. Our findings align with a previous study reporting that a high reaction rate enhances the stochasticity of reaction and precipitation processes, leading to the dislocation of Liesegang-type structures.^71^ In other words, the high concentration of reactants in our study likely contributed to an increased reaction rate, thereby enhancing the randomness of precipitation in the reaction space. For further experimental validation in HS cells, effects of the initial inner electrolyte ([K_2_CrO_4_]_0_) were investigated, varying from 0.1 to 0.5 M with the fixed initial outer electrolyte concentration ([CuCl_2_]_0_) at 1.0 M (Figure 2b). The periodic structures were observed in all conditions except [K_2_CrO_4_]_0_ = 0.1 M. While there were no remarkable changes in the morphology of the pattern when the outer electrolyte concentration was varied, the inner electrolyte strongly influenced it. Namely, an interval distance between successive bands decreased as [K_2_CrO_4_]_0_ increased, and the number of defects increased. With regard to the defect increment, this result is consistent with the above discussion about the role of stochasticity in the precipitation phenomenon.

**Figure 2:**
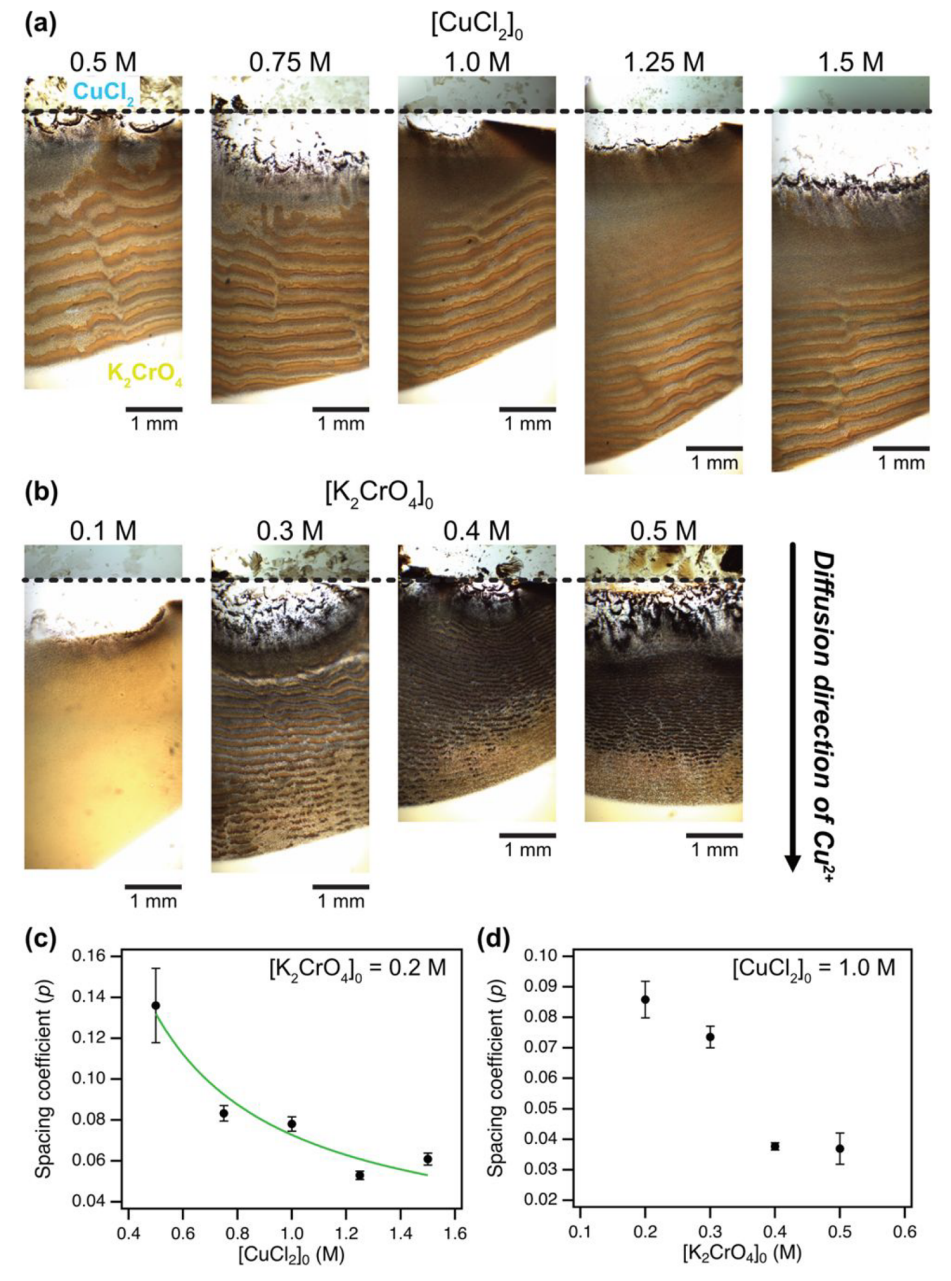
Periodic patterns of CuCrO_4_ precipitates and spatial properties. (a) Optical micrographs of obtained periodic patterns varying an initial concentration of outer electrolyte ([CuCl_2_]_0_) from 0.5 to 1.5 M, where an inner electrolyte initial concentration ([K_2_CrO_4_]_0_) was fixed at 0.2 M. (b) Obtained periodic patterns varying [K_2_CrO_4_]_0_ from 0.1 to 0.5 M, while [CuCl_2_]_0_ was fixed at 1.0 M. The dashed line indicates the edge of the HS cell. All experiments were performed with the HS cell with *d* = 70 μm. The plot of spacing coefficient (*p*) as a function of the initial concentration of (c) CuCl_2_ and (d) K_2_CrO_4_ obtained by using image analysis of all images in (a) and (b) using the ImageJ software. The green curve shows the best fitting to the Matalon–Packter law. Error bars were obtained from 3 replicates calculated using *p*-values <0.05.

To characterize the properties of the obtained periodic structures, we employed the spacing law (eq 1) and the Matalon–Packter (MP) law. These well-known empirical laws are commonly used to evaluate the spatial characteristics of LPs in terms of spacing coefficient (*p*).^45,61^ The MP law is expressed as follows:
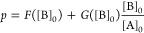
2where [A]_0_ and [B]_0_ are the initial concentrations of the outer and inner electrolytes, respectively. Also, *F* and *G* are monotonously decreasing functions of [B]_0_, which depend on the chemical systems and conditions used. According to this equation, it is straightforward to understand that *p* gradually decreases with increasing [A]_0_ if [B]_0_ is fixed. On the other hand, the effect of [B]_0_ on *p* is complicated because it is included in both decreasing functions. Figure 2c shows the alternation of the spacing coefficient as a function of the initial concentration of the outer electrolyte with the fixed inner electrolyte concentration. The value of *p* was calculated as an average based on the last three bands in Figure 2a using eq 1. The *p* decreased gradually with [CuCl_2_]_0_, and this observed relationship agrees with the MP law prediction as indicated by the green fitting curve. Therefore, the patterns formed in the HS cell in this study can be classified as LPs, and it directly proves that the Liesegang phenomena can be driven not only in solid gel media but also in a liquid phase. Also, a dependence of the inner electrolyte concentration ([K_2_CrO_4_]_0_) on the spacing coefficient was investigated based on photographs in Figure 2b using the same analysis method (Figure 2d). Interestingly, the *p* was decreased against [K_2_CrO_4_]_0_, which was opposite to the previously reported results.^18,42^ The effect of inner electrolyte concentration depends on how the decreasing functions behave. Therefore, the resulting functions could be quite different from previous cases.

To better understand the properties of the observed LPs in the HS cell, we investigated the spatiotemporal dynamics of pattern formation using another widely recognized empirical law for Liesegang phenomena, namely the time law.^62,63^ The expression for the time law is as follows:
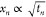
3Here, *t*_n_ represents the time taken to form the *n*th band. The time law is grounded in observing many reaction–diffusion systems in which the characteristic distance in diffusion is linearly proportional to the square root of time. This straightforwardly reflects the diffusive nature inherent in Liesegang phenomena. Figure 3a shows snapshots of time laps during the specific period of pattern formation (*t* = 789–914 s) at [CuCl_2_]_0_ = 1.0 M and [K_2_CrO_4_]_0_ = 0.2 M (for the video of pattern formation made by compression of snapshots, see Video S1). A new precipitate band formed at *t* = 789 s, which the black arrow indicates. This band grew and elongated at a macroscopic scale over time. Then, a depletion zone (the interband region), containing less precipitated particles than within the banding zone, started to form in front of the band when the above growing process was almost completed (*t* = 845 s in Figure 3a). Finally, the next band appeared after the completion of the interband region formation. Accordingly, we observed the alternating formation of bands and depletion zones in a successive pattern as the diffusion and precipitation fronts moved forward. This observation is consistent with the prenucleation mechanism, one of the well-known theories describing pattern formation in Liesegang phenomena.^30,46,47^ Based on the micrograph snapshots, the dynamics of the patterning process were quantified by the time law (Figure 3b). The *x*_*n*_ was entirely linearly proportional to the square root of *t*_*n*_, indicating that the dynamics of LP formation in the HS cell were governed by diffusion aligned with the nature of LPs. Therefore, it was found that the Liesegang phenomenon of the CuCrO_4_ precipitation system in the HS cell was satisfied with the scenario of Liesegang phenomena in solid hydrogels in terms of not only morphological perspectives but also pattern formation dynamics. Also, as another method to evaluate morphologies of LPs, the width law is occasionally used in some previous studies.^64,65^ It is expressed as follows:
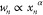
4where *w*_*n*_ is the width of the *n*th band; additionally, the exponent α represents a characteristic coefficient determined by mass conservation for precipitation. Specifically, α tends to approach unity when all the inner electrolyte transforms into precipitate, and the precipitate accumulates only in bands with a constant density.^72^ Conversely, α becomes less than unity either in the presence of unreacted (untransformed) electrolytes or if some electrolytes precipitate at interband positions. Figure 3c displays the plot of *w*_*n*_ versus *x*_*n*_. The value of *w*_*n*_ gradually increased as the band formed away from the interface between the outer and electrolytes, following the nature of the width law. To estimate the exponent α, the plot was converted to a log–log plot using the natural logarithm, and the linear fitting was performed (Figure 3d). The obtained α under this condition was 0.49, noticeably deviating from unity. This result aligns with the previously explained interpretation, suggesting that some precipitated particles are present not only within bands but also in the interband regions of our discovered system (Figure 3a). We performed optical and scanning electron microscopy (SEM) measurements to investigate the size of the formed particles. We obtained that the size of the particles was between 2 and 10 μm, indicating that they did not entirely block the diffusion of the reagents (Figures S2 and S3). In summary, we have demonstrated that CuCrO_4_ LPs can be formed even in a confined liquid layer, and their spatiotemporal properties were utterly consistent with the typical empirical laws of the Liesegang phenomenon. It implies that the essential requirement of reaction media for this phenomenon is low fluidity, eliminating any convection effects, and it is not limited to using solid hydrogels.

**Figure 3:**
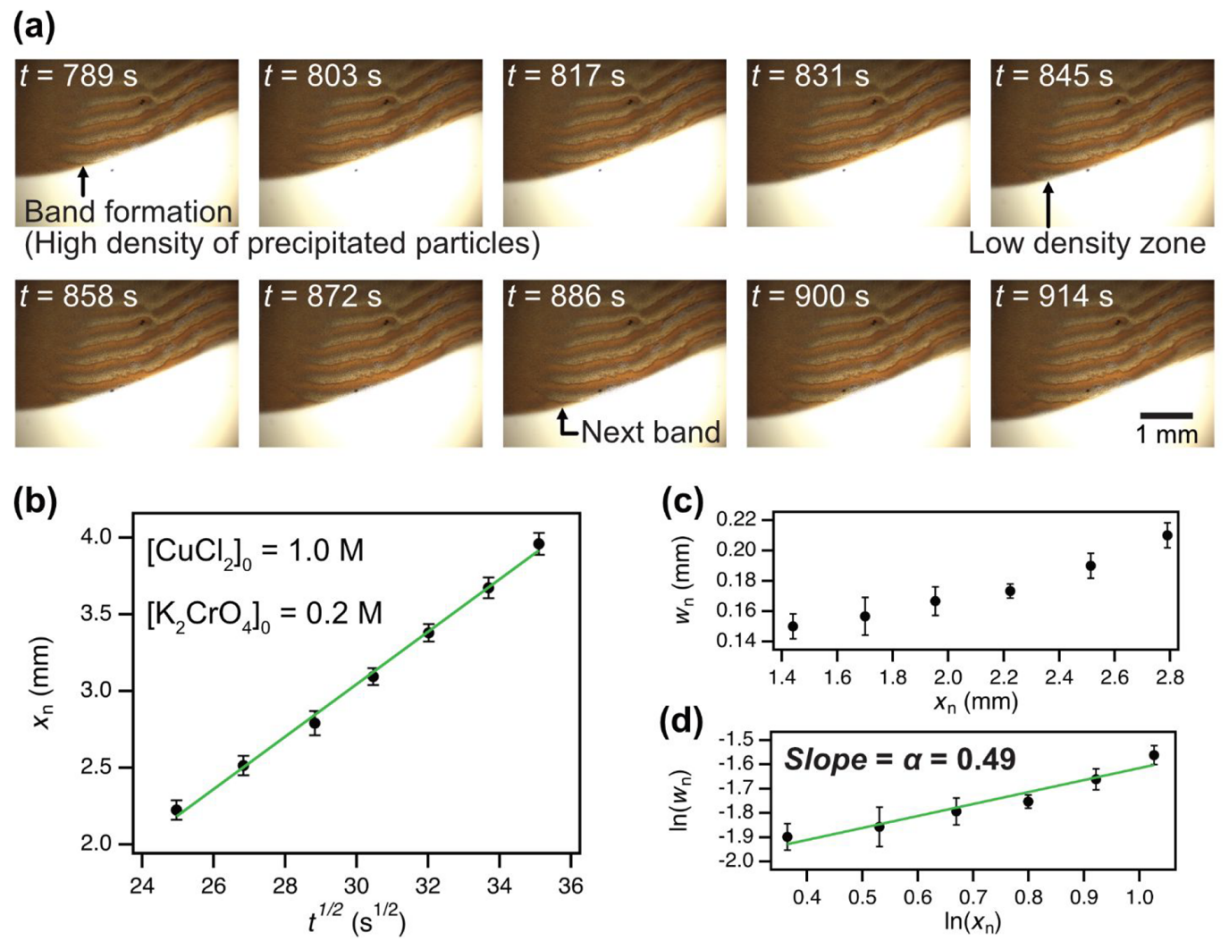
Time course of pattern formation and analysis of the time and width law. Snapshots of time laps during pattern formation ([CuCl_2_]_0_ = 1.0 M, [K_2_CrO_4_] = 0.2 M, and *d* = 70 μm). (a) Processes for forming a precipitation band with a high density of precipitated particles and a depletion zone with a low density of particles took place separately. (b) Plot of the position of the *n*th band (*x*_*n*_) against the square root of time (*t*^1/2^), where *x*_*n*_ corresponds to the distance from the interface between liquid phases of outer and inner electrolytes to each formed precipitated band. (c) Relationship between the width of the nth band (*w*_*n*_) and *x*_*n*_. (d) Plot of the natural logarithm of *w*_*n*_ as a function of the natural logarithm of *x*_*n*_. Green lines show the linear fitting (coefficient of determination: *R*^2^ = 0.999 in (b) and 0.943 in (d)). Error bars were obtained from 3 replicates calculated using *p*-values <0.05.

To extend this concept, we explored other precipitation reaction systems, namely, Ag_2_Cr_2_O_7_^30,44,53^ and zeolitic imidazolate framework-8 (ZIF-8),^26,27,29^ which have been well-investigated for Liesegang-type experiments so far from both experiments and numerical simulations. As the first additional proof, the same experiments in the HS cell (*d* = 70 μm) were carried out, replacing CuCl_2_ and K_2_CrO_4_ with 2.0 M of AgNO_3_ (outer electrolyte) and 0.5 M of K_2_Cr_2_O_7_ (inner electrolyte), respectively (Figure 4a). It was observed that there was no periodic pattern, and the needle-like crystals were formed in the HS cell entirely when the aqueous phase contained only electrolytes (Figure 4a, left). Some previous studies reported that Ag_2_Cr_2_O_7_ could form LPs in gelatin gels, while random crystallites were formed in agarose gels.^43,44^ These studies also observed a transition in pattern morphologies toward periodic shapes by adding a small amount of gelatin to agarose gels, with the periodicity increasing as more gelatin is added. Scanning electron microscopy measurements from these studies revealed that gelatin exhibited well-organized microporous structures with pores approximately 3–4 μm in diameter, whereas agarose provided densely packed flakes without specific micrometric constituents.^43^ It suggests that the gelatin can facilitate nucleation effectively because it can offer a larger surface area that can act as a potential nucleation site. Accordingly, even in our system, it was expected that adding a small amount of gelatin into the aqueous phase of the inner electrolyte probably improved the system, leading to more periodicity in pattern morphologies rather than the formation of needle-like crystals. A structural anisotropy of formed particles got less. It seemed more amorphous when 2 × 10^–3^% w/v of gelatin was added (Figure 4a, second panel), but there were still no macroscopic periodic structures. However, when the added gelatin was increased more than 5 × 10^–3^% w/v (Figure 4a, third and fourth panels), the transition of pattern morphologies toward the periodic structure (Liesegang patterns) was observed. Additionally, the size of the precipitated particles became smaller, and their shape was almost spherical. It supported that the nucleation dominated precipitation processes more than the crystal growth, which was facilitated by the presence of gelatin. It must be noted that the phase containing gelatin was liquid at room temperature with these gelatin concentrations. As explained previously, many researchers have tried controlling LP morphologies with gel-based tuning methods.^16,31^ The numerical simulations showed that nucleation rate should be a key factor.^73,74^ However, gels could also affect other phenomena, such as diffusivity of ions, precipitation, sedimentation, and hydrodynamic stabilities. In contrast, the medium in our developed system consists entirely of liquid, and the gelatin added is relatively low. The gelatin critically contributed to changing the morphology of the pattern. It indicates that our system is appropriate to demonstrate the impact of nucleation rate on pattern morphologies, and it was found that nucleation is crucial to modulate LPs. Previous studies showed that the precipitate of Ag_2_Cr_2_O_7_ in gelatin hydrogel has a core–shell structure with a metallic (silver) core.^75−77^ Based on this finding, and knowing that gelatin can reduce silver ions (generating nanoparticles),^78^ the underlying mechanism of the LP formation in this system can be the heterogeneous nucleation facilitated precipitation of Ag_2_Cr_2_O_7_ driven by the presence of silver nanoparticles. However, our results show that the heterogeneous nucleation centers cannot be silver nanoparticles because the time (several minutes), low gelatin concentration, and room temperature cannot produce silver nanoparticles. We hypothesized that the nucleation centers facilitating the precipitation process consist of complexes of silver ions with gelatin components (amino acids). It should be noted that after the formation of the LP, the amino acids can reduce the silver ions in the cores of the particles at the characteristic time scale of the pattern formation (several days).

**Figure 4:**
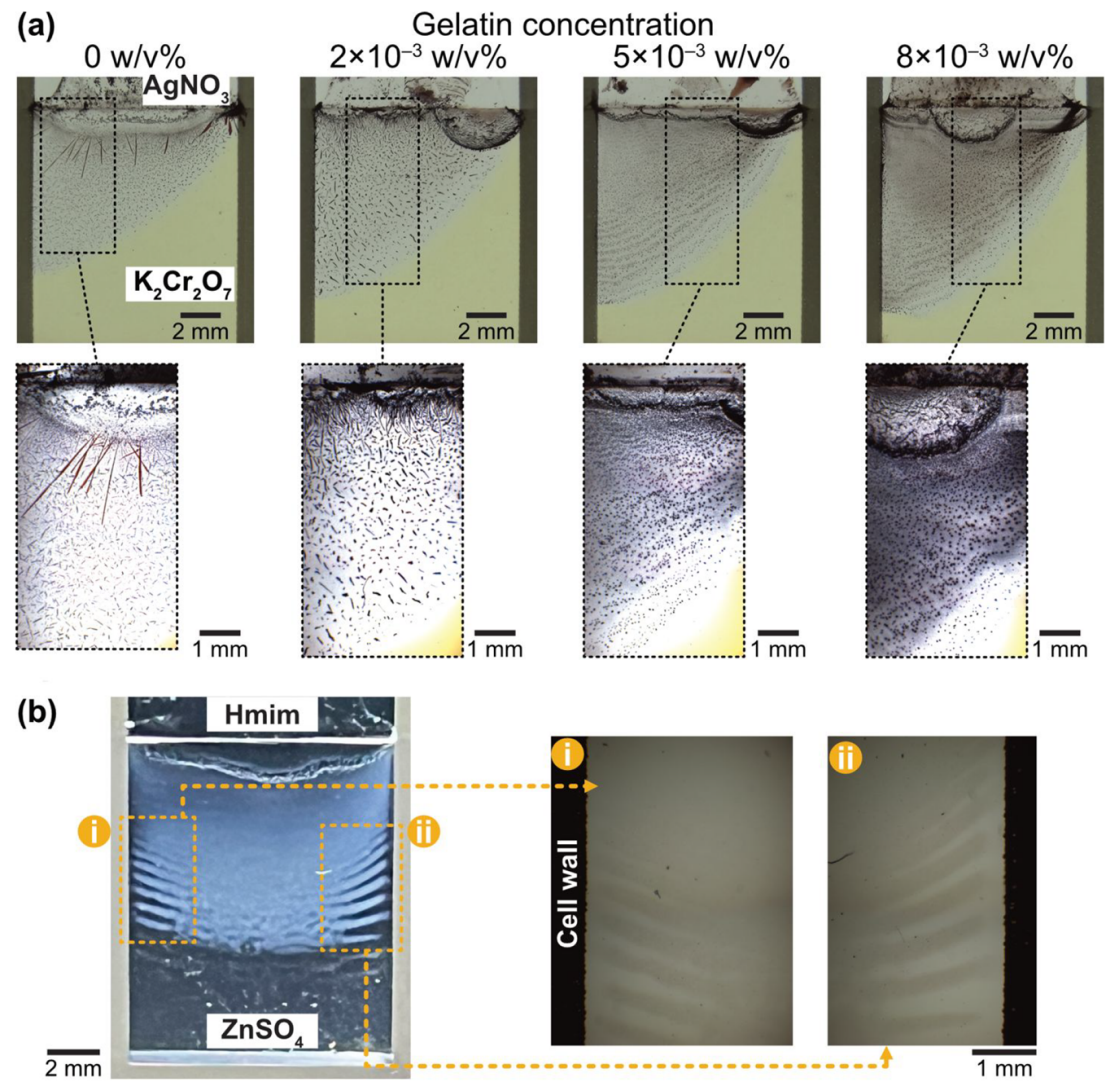
Pattern formation in different precipitation reaction systems (Ag_2_Cr_2_O_7_ and ZIF-8). (a) Photographs and micrographs of morphology transition of Ag_2_Cr_2_O_7_ precipitation patterns varying the gelatin concentration from 0 to 8 × 10^–3^% w/v. In all cases, 2.0 M of AgNO_3_ and 0.5 M of K_2_Cr_2_O_7_ were used as outer and inner electrolytes, respectively. Each micrograph was obtained from a region surrounded by a broken line square in each photograph. (b) Photograph and micrographs of ZIF-8 precipitation pattern for 1.0 M of Hmim (outer electrolyte) and 0.01 M of ZnSO_4_ (inner electrolyte), where all solutions were made in a mixture of water and DMF (1:1 volume ratio). All experiments were conducted in a HS cell with *d* = 70 μm.

From the material science point of view, materials are expected to have some applications, namely, a ZIF-8 precipitation system, which were also investigated other than the classic insoluble salt systems demonstrated above (Figure 4b and Figure S4). In this experiment, 2-methylimidazole (Hmim) and ZnSO_4_ were used as outer and inner electrolytes dissolving in a mixture of water and *N*,*N*-dimethylformamide (DMF) with a 1:1 volume ratio. The periodic precipitation of ZIF-8 appeared near the edge of the cell wall when [Hmim]_0_ = 1.0 M and [ZnSO_4_] = 0.01 M were used (Figure 4b). Furthermore, a similar periodic pattern was observed when the initial concentration of ZnSO_4_ increased up to 0.1 M (Figure 4b). These results prove that Liesegang phenomena can be engineered using a HS cell in various precipitation reaction systems.

We have successfully demonstrated the formation of LPs in a HS cell having 70 μm of the vertical gap distance (*d*). As the final part of this study, we directed our attention to understanding the impact of *d* on Liesegang phenomena, aiming to gain deeper insights into the role of HS cells in this context. From a fluid physics perspective, precipitation is expected to induce turbulent convection driven by the density difference of fluids (buoyancy effect) along with sedimentation of colloidal particles if the gap in HS cells exceeds a critical value. This is because a viscous force restraining flow becomes less significant than fluid inertia (fluid momentum). Therefore, if the utilized HS cell gap (*d* = 70 μm) is less than the critical gap, it provides direct evidence that the fluid instability inducing convection can be adequately restricted by a sufficiently confined liquid reaction space similar to gels. This confinement facilitates the formation of LPs in a liquid phase. To enable us to investigate the effects of *d* experimentally, we crafted another HS cell by inserting a layer of paraffin polymer films with different numbers between two glass plates (Figure S5a). In this way, the value of *d* was certainly adjusted proportionally to the number of layers (Figure S5b). Experiments of pattern formation with the CuCrO_4_ system ([CuCl_2_]_0_ = 1.0 M and [K_2_CrO_4_] = 0.2 M) were performed with the crafted HS with different *d* values that were greater than 70 μm (Figure 5a). The LP was observed only at *d* = 120 μm. Otherwise (*d* = 260 and 680 μm), the homogeneous distribution of the precipitate could be observed, and it can no longer be classified as a periodic structure. Thus, the critical gap was located between 120 and 260 μm. To confirm the validity of this experimental observation from a fluid physics standpoint, we calculated the Rayleigh–Darcy number, considering the effects of the geometry of HS cells (ε^2^*Ra*).^66−69^ This calculation estimates the relative strength of convection to diffusion. The Rayleigh–Darcy number (*Ra*) and the geometry parameter (ε) can be calculated separately based on the following equations:
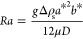
5
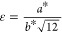
6where *g*, a*, b*, μ, and *D* are the standard acceleration of gravity, the smallest length of the cell, the length of the cell parallel with the gravity acceleration vector, the dynamic viscosity of the medium, and the molecular diffusion coefficient, respectively. Also, Δρ_s_ is the density difference between solute-saturated fluids and solute-free fluids. In our setup, both a* and b* equal *d*, and eqs 5 and 6 can be rewritten in the form
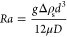
7
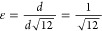
8

**Figure 5:**
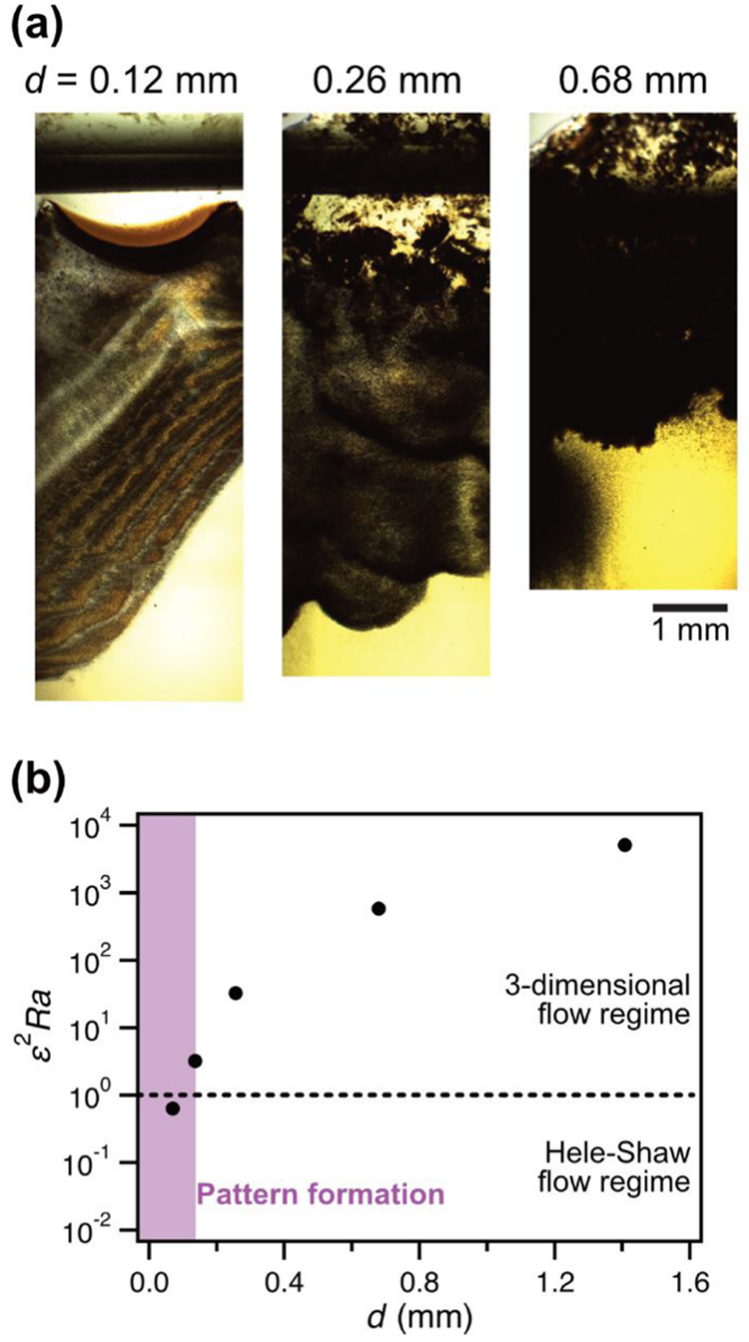
Effect of vertical gap distance (*d*) on the pattern formation of CuCrO_4_ system in both the HS cell and a hand-crafted HS cell. (a) Pattern formation in the hand-crafted HS cells with different *d* values for [CuCl_2_]_0_ = 1.0 M and [K_2_CrO_4_]_0_ = 0.2 M: *d* = 0.12, 0.26, and 0.68 mm. (b) Variation of the Rayleigh–Darcy number with the cell anisotropy ratio (ε^2^*Ra*) as a function of *d*. The purple square indicates the region where the periodic pattern was formed. The dotted line corresponds to the 2D HS and 3D flow regimes boundary.^68,69^ Error bars were obtained from 3 replicates calculated using *p*-values <0.05.

The detailed parameters and results of the calculation with different *d* values are shown in Table S1. Figure 5b plots the calculated ε^2^*Ra* values versus *d*, including commercial and hand-crafted HS cells. The previous studies reported that ε^2^*Ra* serves as a discriminator between two distinct flow configurations: (i) Hele–Shaw regime (ε^2^*Ra* ≪ 1), characterized by two-dimensional flow influenced by gap-induced dispersion, and (ii) three-dimensional regime (ε^2^*Ra* ≥ 1), where the effects of the third dimension become non-negligible.^68,69^ In our investigation, we observed that the last appearance of the periodic structure with the increasing cell gap occurred at a value (ε^2^*Ra* = 3.2 in the case of *d* = 120 μm), which is close to the value corresponding to the boundary between these two flow regimes (ε^2^*Ra* = 1), and the LPs were observed at the regime of Hele–Shaw flow. This observation underscores the critical role of HS cells in effectively controlling the fluidity of confined liquid phases. It aligns with predictions based on fundamental fluid physics theories, indicating that HS cells can play a role similar to gels in Liesegang phenomena.

In conclusion, we have demonstrated that LPs of the CuCrO_4_ precipitation system were generated in the liquid layer confined into the HS cell having a width gap of 70 μm. Notably, the spatiotemporal properties of obtained LPs were perfectly consistent with well-known empirical laws of LPs, such as the spacing, MP, time, and width laws. Furthermore, LPs appeared not only in the CuCrO_4_ system but also in other precipitation systems, including Ag_2_Cr_2_O_7_ and ZIF-8. Adding a small amount of gelatin to the Ag_2_Cr_2_O_7_ system effectively facilitated the morphological transition between the random crystalline formation and periodic pattern formation at the macroscale. This result should highlight the role of nucleation in the Liesegang phenomena without considering any physics and chemical complicated effects of gels. Then, we experimentally investigated the condition of the critical gap distance of HS cells (*d*) where the LPs were formed using the hand-crafted HS cell. It was finally found that the critical *d* was entirely in agreement with the simple prediction based on the Rayleigh–Darcy number, considering the effects of the geometry of HS cells (ε^2^*Ra*). Therefore, this study has demonstrated that the presence of a gel is not essential for the Liesegang phenomenon. Instead, the fluidity of the reaction medium, extending beyond gel or liquid phases, is a crucial factor capable of effectively limiting hydrodynamic instability. This finding emphasizes the need to consider fluidity as a significant factor in the Liesegang phenomena. This discovery introduces increased diversity into experiments related to the Liesegang phenomena. By showing a breakthrough that challenges the classical experimental setups, in which only gels have been used as reaction media for over a century since the discovery of the phenomenon, we have paved the way for new approaches in this field. A more complex approach could be the investigation of the formation of periodic precipitation in a wedge-shaped HS. This setup affects not only the fluidity of the medium but the diffusion flux of the chemical species, contributing to the creation of more complex chemical structures in reaction–diffusion systems. Additionally, the demonstration of pattern formation in a gel-free medium, entirely in solution, is anticipated to expedite research on material synthesis, especially metal nanoparticles and MOFs, using this phenomenon.^25−29^ This study area has already seen extensive reporting in recent years, and our findings are poised to contribute significantly to its advancement.

## Supporting Information Available

Description of the experiments, Figures S1–S5 showing the details of the used HS cell, photos of ZIF-8 precipitation in the HS cell with different concentrations of ZnSO_4_, optical and SEM micrographs of the CuCrO_4_ particles, design of hand-crafted HS cell, and Table S1 presenting parameters and results for calculating the Rayleigh–Darcy number and the geometry parameter. (PDF)
Video S1: pattern formation in the CuCrO_4_ system (MP4)
Transparent Peer Review report available (PDF)
