# Supplementary material for: Periodic Precipitation in a Confined Liquid Layer
Source: J Phys Chem Lett. 2024 Apr 30;15(18):4948–57. doi: 10.1021/acs.jpclett.4c00832 (PMC11089569; doi:10.1021/acs.jpclett.4c00832)
Supplement: Supplementary file 1 — jz4c00832_si_001.pdf [file jz4c00832_si_001.pdf]

Supporting Information

Periodic Precipitation in a Confined Liquid Layer

Masaki Itatani,[1*] Yuhei Onishi,[2] Nobuhiko J. Suematsu,[2, 3] István Lagzi[1,4*]

[1]Department of Physics, Institute of Physics, Budapest University of Technology and Economics, Budapest H-1111, Hungary

[2]Graduate School of Advanced Mathematical Sciences, Meiji University, Tokyo 164-8525, Japan

[3]Meiji Institute for Advanced Study of Mathematical Sciences (MIMS), Meiji University, Tokyo 164-8525, Japan

[4]HU-REN–BME Condensed Matter Physics Research Group, Budapest University of Technology and Economics, Budapest H-1111, Hungary

1. Experimental

1.1 Pattern formation and analysis

In typical experiments, a commercially sold HS cell constitutes two parallel transparent glass plates separated by a narrow space (SEKISUI CHEMICAL Co., Ltd., UR–157–S) was used (Figure S1a). The size of the cell window is 10 × 12 mm, and the vertical gap distance between a cover glass and bottom glass substrate (d) is 70 μm. The stock solutions of each metal ion, electrolyte, and linker were prepared with the given concentration: 1.5 M of copper (II) chloride ($CuCl_2$, Sigma-Aldrich, 97%), 0.5 M of potassium chromate (K_2CrO_4, Sigma-Aldrich, ≥99.0%), 2.0 M of silver nitrate ($AgNO_3$, Sigma-Aldrich, ≥99.8%), 0.5 M of potassium dichromate ($K_2Cr_2O_7$, Renal, analytical grade), 0.2 M of zinc sulfate heptahydrate ($ZnSO_4 \cdot 7H_2O$, Sigma-Aldrich, ≥99.0%), and 2-methylimidazole (Hmim, Sigma-Aldrich, 99%). Only solutions of $ZnSO_4$ and Hmim were made by a mixture of ultra-purified water and *N*,*N*-dimethylformamide (DMF, Sigma-Aldrich, ≥99%) with a 1:1 volume ratio, while other solutions were made by dissolving electrolytes in ultra-purified water. For the procedures of pattern formation, the cell window was filled with an inner electrolyte solution first. Then, 20 μL of an outer electrolyte solution was carefully contacted with the inner electrolyte or linker solution at the edge of the cell window (Figure S1b). Immediately after contacting these two solutions, preferential diffusion of outer electrolytes was started because of higher concentration than the inner electrolytes, and precipitation reactions occurred in the cell. The process of pattern formation was observed at room temperature (~25 °C), and snapshots were taken for 15 min with 10-15 s intervals by a microscope (VWR, VisiScope TL324H) equipped with a CCD camera (VWR,

VisiCam 10.0). Obtained photographs and micrographs were analyzed by the ImageJ software to investigate the spatiotemporal properties of obtained patterns.

1.2 Craft of an HS cell using paraffin film layers

Stacked paraffin films (BRAND, Seal-R-film) with different numbers of layers, which were cut with the same shape and geometry as the above HS cell, were inserted in between two slide glasses (Thermo Scientific, Microscope slides) (Figure S3a). Subsequently, it was heated until the films started to melt, and it was cooled out at room temperature immediately after this to solidify the films and stick them to glasses completely. The pattern formation experiments were carried out with the same procedure explained above.

1.3 Sample preparation for the scanning electron microscopy (SEM)

The solution of K_2CrO_4 (0.2 M, 5 μL) was sourced into the narrow space between the slide glass and cover glass, and then, $CuCl_2$ aqueous solution (1.0 M, 3 μL) was added. After the pattern formation, the reactant solutions were removed by blotting paper, and the cover and slide glasses were separated and dried at ambient conditions. Gold sputtering was performed to maintain appropriate electrical conductance. The morphology of precipitates was investigated using a scanning electron microscope (Phenom proX PREMIUM, Phenom World).

Video S1. Periodic precipitation pattern formation in a liquid phase in copper chromate systems ($[CuCl_2]_0$ = 1.0 M, $[K_2CrO_4]_0$ = 0.2 M, and d = 70 μm). The video plays for 125 seconds and has a domain size of 4.0 × 3.0 mm.

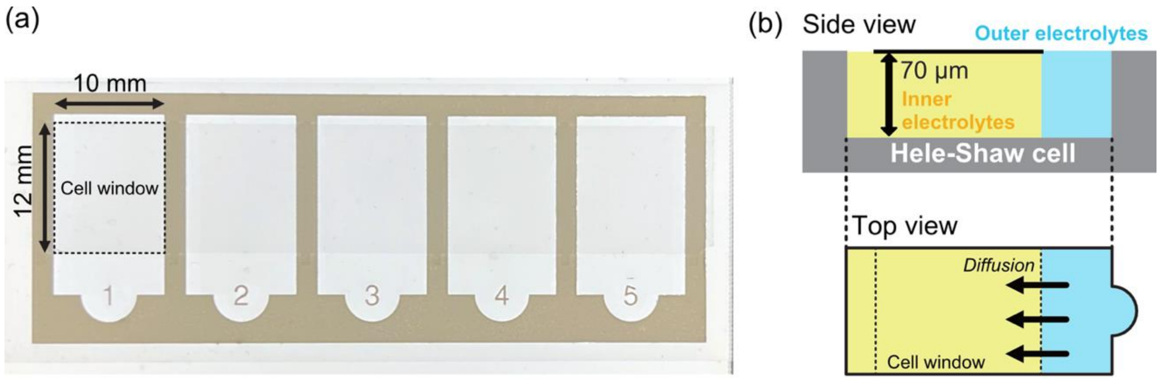

Figure S1. Experimental setup. (a) Hele-Shaw cell was used in this study. The pattern formed inside a cell window indicated by a broken line square. (b) Sketch of how to initiate pattern formation.

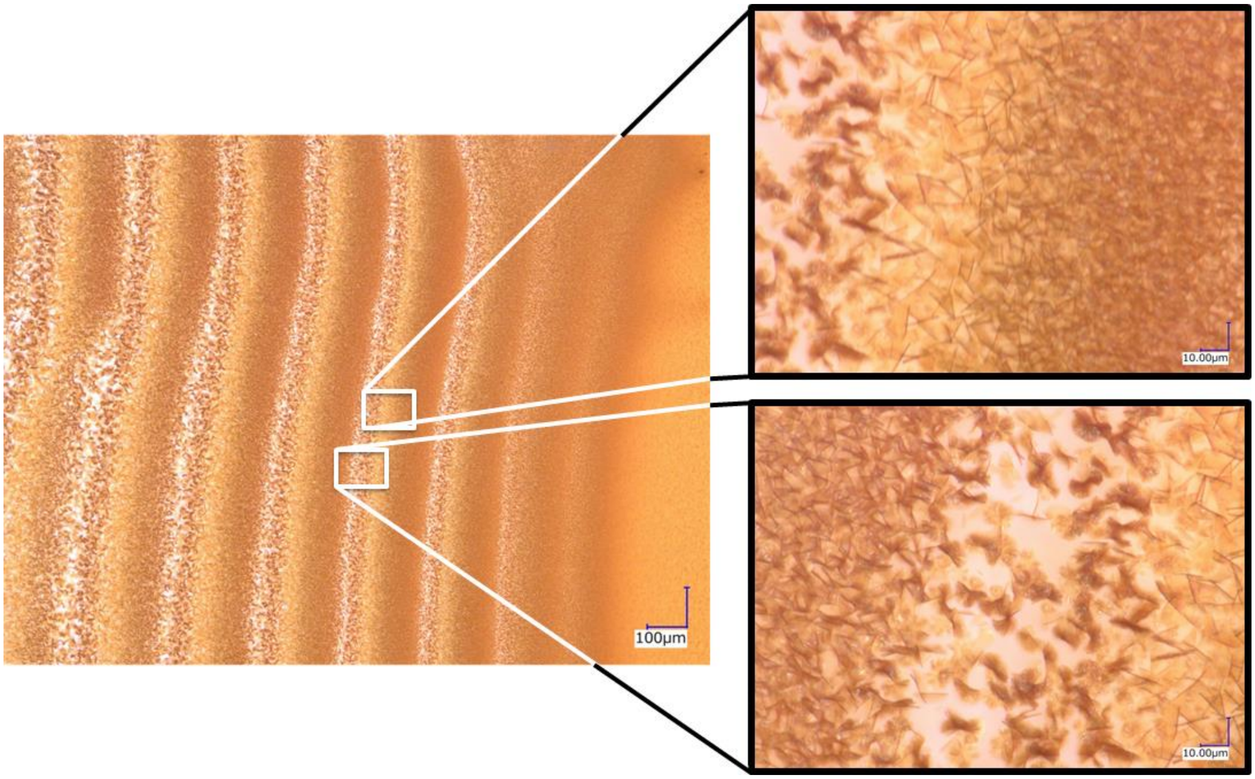

Figure S2. Optical micrographs of the pattern formed in the HS cell with d = 70 μm under the following conditions: $[CuCl_2]_0$ = 1.0 M and $[K_2CrO_4]_0$ = 0.2 M.

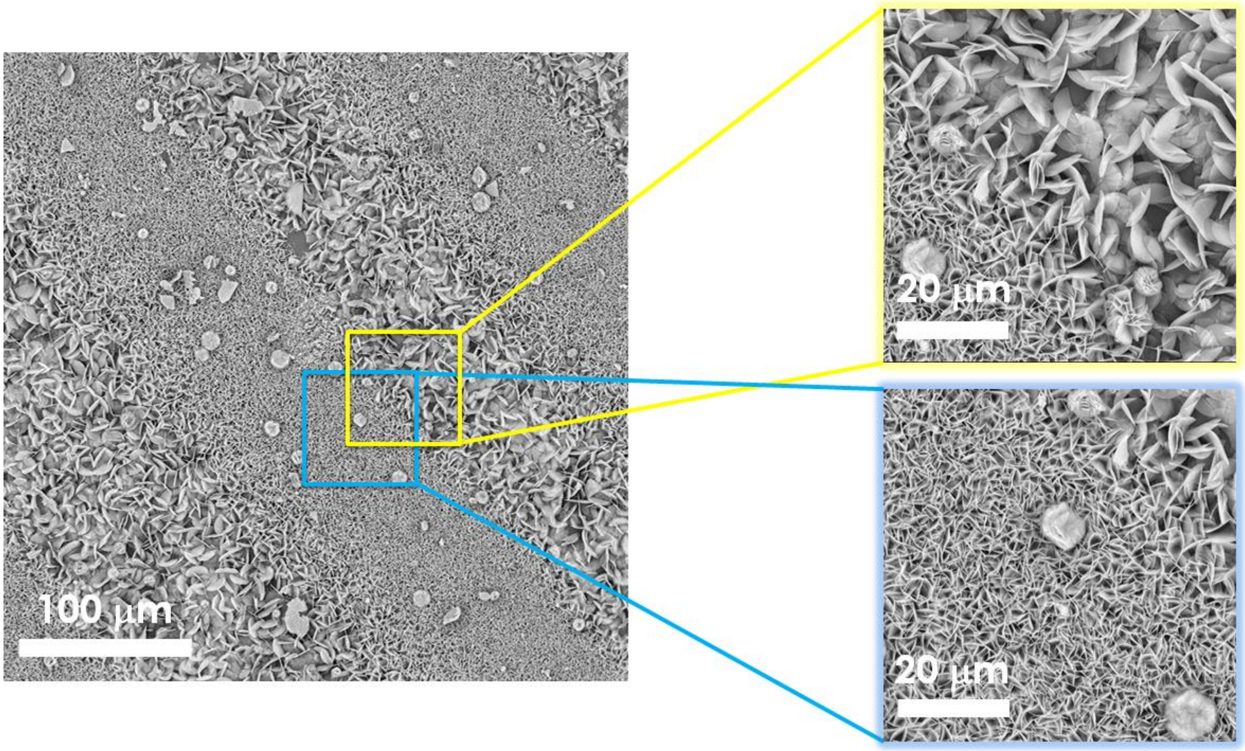

Figure S3. SEM micrographs of the pattern formed in the bottom glass slide of the HS cell with *d* = 70 μm under the following conditions: $[CuCl_2]_0$ = 1.0 M and $[K_2CrO_4]_0$ = 0.2 M.

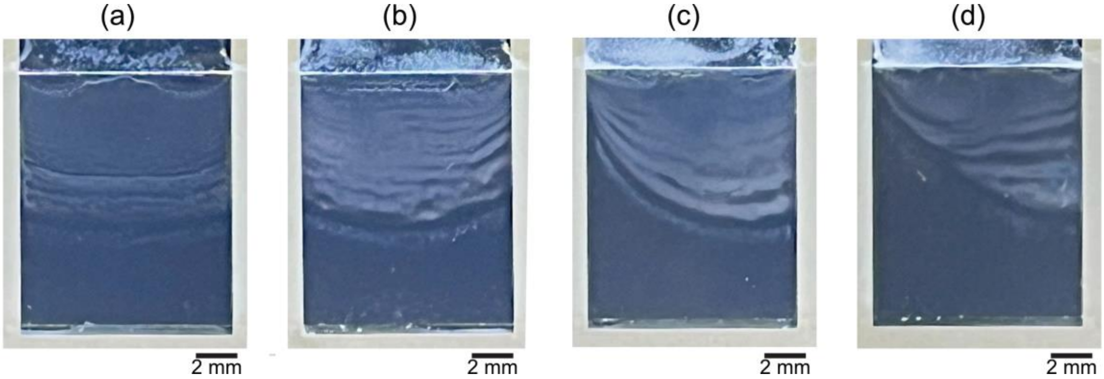

Figure S4. Photographs of ZIF-8 precipitation. Each image corresponds to experiments carried out in varying $[ZnSO_4]_0$ concentrations: (a) 0.02 M, (b) 0.05 M, (c) 0.1 M, and (d) 0.2 M. In all cases, $[Hmim]_0$ was fixed at 1.0 M.

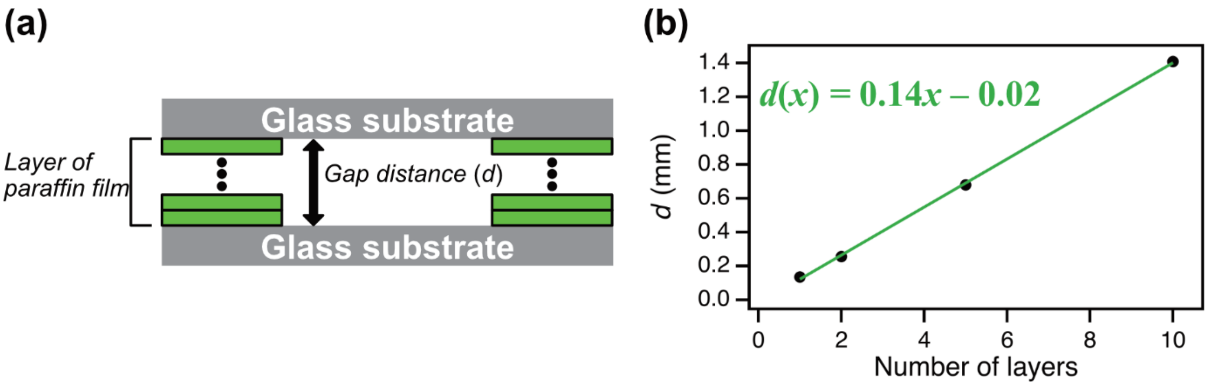

Figure S5. (a) Illustration of hand-crafted HS cell, where *d* can be tuned by changing the number of paraffin film layers. (b) Relationship between *d* and the number of spacer layers. The green line shows the linear fitting (R^2 = 0.999).

Table S1. Parameters and results for calculating the Rayleigh-Darcy number (*Ra*) and the geometry parameter (ε).

d (m)	g (m s^{-2})	$\Delta\rho_s$ (kg m^{-3})[a]	D (m^2 s^{-1})[b]	μ (Pa s)[c]	Ra	$\varepsilon^2 Ra$
7.0×10^{-5}	9.8	27	1.0×10^{-9}	9.9×10^{-4}	7.6	6.4×10^{-1}
1.2×10^{-4}					3.9×10^{1}	3.2×10^{0}
2.6×10^{-4}					3.9×10^{2}	3.2×10^{1}
6.8×10^{-4}					7.0×10^{3}	5.8×10^{2}
1.4×10^{-3}					6.1×10^{4}	5.1×10^{3}

[a] $\Delta\rho_s$ was calculated as follows: $\Delta\rho_s = \rho_s - \rho_0$, where ρ_0 is the density of solution only containing 0.2 M of K_2CrO_4, and ρ_s is the density of solution containing $CuCrO_4$ precipitates and counter ions of outer and inner electrolytes (Cl^- and K^+) estimated by assuming that all K_2CrO_4 is transformed to $CuCrO_4$.

[b] The typical value of metal ions was used as D in this study, but it is very similar to the actual value of Cu^{2+} in aqueous solutions.[1]

[c] μ was calculated based on the previous study.[2]

References

(1) Ribeiro, A. C. F.; Esteso, M. A.; Lobo, V. M. M.; Valente, A. J. M.; Simões, S. M. N.; Sobral, A. J. F. N.; Burrows, H. D. Diffusion Coefficients of Copper Chloride in Aqueous Solutions at 298.15 K and 310.15 K. *J. Chem. Eng. Data* **2005**, *50*, 1986–1990.

(2) Jones, G.; Colvin, J. H. The Viscosity of Solutions of Electrolytes as a Function of the Concentration. VII. Silver Nitrate, Potassium Sulfate and Potassium Chromate. *J. Am. Chem. Soc.* **1940**, *62*, 338–340.
