# Supplementary material for: Periodic Precipitation in a Confined Liquid Layer
Source: J Phys Chem Lett. 2024 Apr 30;15(18):4948–57. doi: 10.1021/acs.jpclett.4c00832 (PMC11089569; doi:10.1021/acs.jpclett.4c00832)
Supplement: Supplementary file 3 — jz4c00832_si_003.pdf [file jz4c00832_si_003.pdf]

jz-2024-00832a.R1

Name: Peer Review Information for "Periodic Precipitation in a Confined Liquid Layer"

First Round of Reviewer Comments

Reviewer: 1

Comments to the Author

The paper by Itatani et al. reports an experiment study of Liesegang patterns in Hele-Shaw cells. HS cells are frequently used for analysis of viscous fingering and its relevance to oil exploration and via Darcy's law flow in porous media. Liesegang patterns (LPs) are usually produced in gels, which defines the main novelty of this paper. The authors avoid undesired fluid motion not by polymers but rather by vertical confinement to a very thin solution layer (typically 70 um) bound by two glass plates. The importance of this study lies at least partially in the possibility of characterizing the role of the polymer/gel on the observed patterns, ultimately providing a better understanding of this classic and interesting phenomenon. I think the study is of high quality and originality. It is also well-written and of interest to physical chemists. I recommend the paper for publication in JPC Letters with minor revisions. I specifically ask the authors to consider the following questions and comments.

1) Title: The current title made me think of small pores and similar three-dimensional spatial confinement. Perhaps (and I do not insist) change "phase" to "layer".

2) P. 18: What is meant by "Otherwise (d = 260 and 680 μm), a tubularlike pattern appeared no longer classified as periodic structures as LPs"? Could this be a chemical garden?

3) Figures 5a,b are not needed and could be moved to the SI. They simply describe how the authors varied the gap height.

4) Eq. 5: I am confused that the cell length L enters the analysis. Would the patterns (onset of LP) be different if cells of say 1 vs 10 cm are used? Also ε^2Ra depends on L! Moreover, microgravity conditions would yield Ra=0, but I doubt that LPs would form in thick layers for g = 0.

5) While I am not requesting these data, it might be interesting to repeat the experiment in a wedge-shaped HS cell with heights between say 20 and 200 um.

6) In the Science paper https://doi.org/10.1126/science.aah6350, the Aizenberg group shows some indication of Liesegang patterns formed by biomorphs in microfluidic devices (see Fig. 1G, see also

the SI). The results are not fully convincing, but might be worth mentioning (or at least of interest to the authors).

7) Can the authors provide more information on the size of the crystals formed in their different systems. Is the crystal height comparable to the layer height? Should we expect differences between a full and partial blockage of diffusion by these crystals?

8) Does the surface (what is the material?) of the HS cell affect crystal growth? Would different materials potentially create different outcomes or do the crystals nucleate in solution?

9) P. 21: rephrase “>empirical stereotypes< of experiments in use”.

10) Check for missing subscripts (e.g. caption 5, SI text at the end of the main paper).

Reviewer: 2

Comments to the Author

This manuscript describes the periodic precipitation patterns using a Hele-Shaw cell in a confined liquid phase. I think that the authors have reported a really interesting phenomenon. In addition to the interesting phenomenon, the authors have also investigated the phenomenon in good amount of depth, both experimentally and theoretically. The conclusion arrived based on the investigations is thus strong. The demonstration of pattern formation in a gel-free environment is indeed interesting. I think this manuscript is very suitable for the Journal of Physical Chemistry Letters.

Author's Response to Peer Review Comments:

István Lagzi
Associate Professor
Budapest University of Technology and Economics
Institute of Physics

H-1111, Budafoki út 8.
Budapest, Hungary
Tel: +36 1463-1341
WWW: nimbus.elte.hu/~lagzi
Email: lagzi.istvan.laszlo@ttk.bme.hu

April 22, 2024

Senior Editor
The Journal of Physical Chemistry Letters

Dear Editor,

Thank you for sending us the very helpful comments on our manuscript entitled ***„Periodic Precipitation in a Confined Liquid Layer”***. In the following, we provide our point-by-point answers to the specific queries. For clarity, the Reviewers' comments and questions are formatted in *italics*.

Manuscript Formatting Request - Non-scientific changes

“2) TOC Graphic: Please resize the TOC graphic per journal guidelines (2 in x 2 in).”

Author reply: We resized the TOC graphic according.

*“3) References: In both the main file and the supporting information, fix the style of all references to use JPCL formatting (check all references carefully). ***JPC Letters reference formatting requires that journal references should contain: () around numbers; author names; article title (titles entirely in title case or entirely in lower case); abbreviated journal title (italicized); year (bolded); volume (italicized); and pages (first-last). Book references should contain author names; book title (in the same pattern); publisher; city; and year. Websites must include date of access.”*

Author reply: We checked the reference style both in the manuscript and supporting information.

“4) Graphics: One or more of your figures and tables includes a reference citation. Please confirm that this pertains only to data and not the figure itself. If it pertains to the use of a published image, permissions must be secured for any graphics NOT originally published by ACS or for Open Access content which permits reuse with credit only. Permission is needed if you are using another publisher's or copyright owner's figures/tables verbatim, adapting/modifying them, or using them in part. If the images are from an Open Access publisher that does not require permission for reuse, please confirm.”

Author reply: We confirm that Table S1 contains two citations that pertain only to data.

<u>Reviewer #1</u>

„The paper by Itatani et al. reports an experiment study of Liesegang patterns in Hele-Shaw cells. HS cells are frequently used for analysis of viscous fingering and its relevance to oil exploration and via Darcy’s law flow in porous media. Liesegang patterns (LPs) are usually produced in gels, which defines the main novelty of this paper. The authors avoid undesired fluid motion not by polymers but rather by vertical confinement to a very thin solution layer (typically 70 um) bound by two glass plates. The importance of this study lies at least partially in the possibility of characterizing the role of the polymer/gel on the observed patterns, ultimately providing a better understanding of this classic and interesting phenomenon. I think the study is of high quality and originality. It is also well-written and of interest to physical chemists. I recommend the paper for publication in JPC Letters with minor revisions. I specifically ask the authors to consider the following questions and comments.”

Author reply: We thank the Reviewer for his/her comments and the positive assessment of our work.

“1) Title: The current title made me think of small pores and similar three-dimensional spatial confinement. Perhaps (and I do not insist) change “phase” to “layer”.”

Author reply: We agree with the Reviewer’s suggestion and have changed the title to “Periodic Precipitation in a Confined Liquid Layer”.

“2) P. 18: What is meant by “Otherwise (d = 260 and 680 μm), a tubularlike pattern appeared no longer classified as periodic structures as LPs”? Could this be a chemical garden?”

Author reply: We rewrote this sentence for clarity. We do not think these patterns can be a chemical garden. The chemical garden involves the formation of tubular structures with a semipermeable property.

Budapest University of Technology and Economics
Faculty of Natural Sciences ● Institute of Physics
Fizika Tanszék

1111 Budapest, Budafoki út 8. F.épület, Hungary
Phone: +36 1463-2312, Fax: +36 1463 4180
http://dept.phy.bme.hu

Changes: Page 18.
Otherwise (d = 260 and 680 μm), the homogeneous distribution of the precipitate could be observed, and it can no longer be classified as a periodic structure.

"3) Figures 5a,b are not needed and could be moved to the SI. They simply describe how the authors varied the gap height."

Author reply: We thank the Reviewer for this comment. We moved Figure 5 a,b to the SI (now Figure S3 in the SI).

Changes:
(i) Page 20.

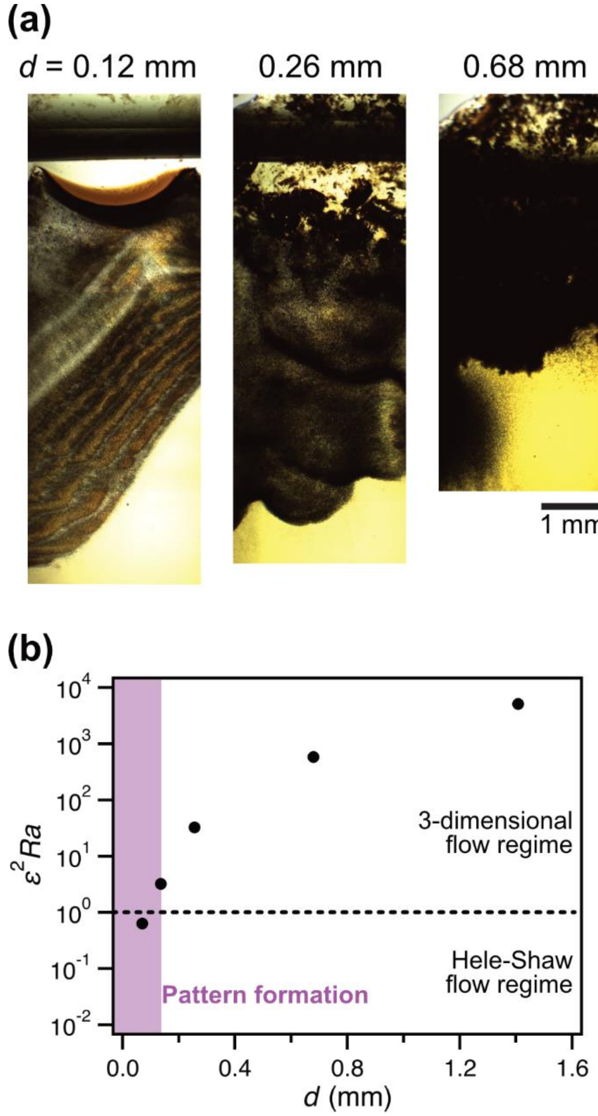

Budapest University of Technology and Economics
Faculty of Natural Sciences • Institute of Physics
Fizika Tanszék
1111 Budapest, Budafoki út 8. F.épület, Hungary
Phone: +36 1463-2312, Fax: +36 1463 4180
http://dept.phy.bme.hu

Figure 5. Effect of vertical gap distance (*d*) on the pattern formation of $CuCrO_4$ system in both the HS cell and a hand-crafted HS cell. (a) Pattern formation in the hand-crafted HS cells with different *d* values for $[CuCl_2]_0$ = 1.0 M and $[K_2CrO_4]_0$ = 0.2 M: *d* = 0.12 mm, 0.26 mm, and 0.68 mm. (b) Variation of the Rayleigh–Darcy number with the cell anisotropy ratio ($\varepsilon^2 Ra$) as a function of *d*. The purple square indicates the region where the periodic pattern was formed. The dotted line corresponds to the 2D HS and 3D flow regimes boundary.[68,69] Error bars were obtained from 3 replicates calculated using *p*-values < 0.05.

(ii) SI

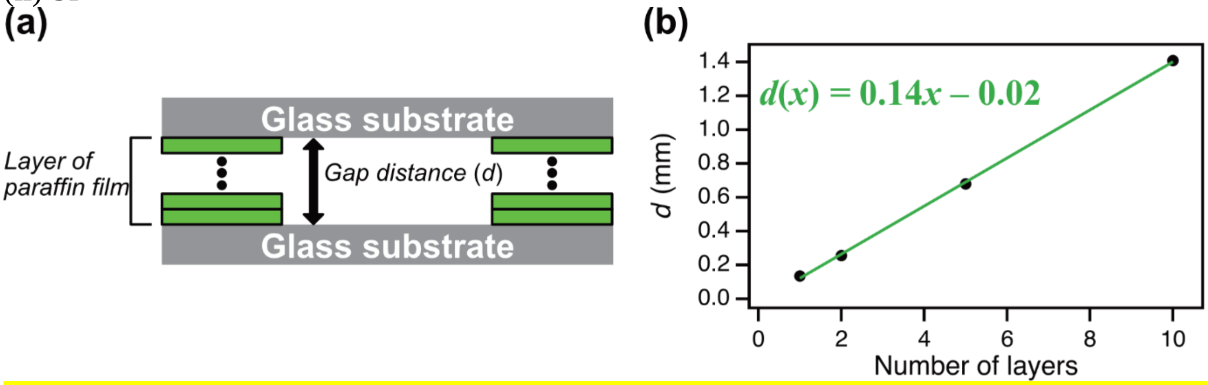

Figure S3. (a) Illustration of hand-crafted HS cell, where *d* can be tuned by changing the number of paraffin film layers. (b) Relationship between *d* and the number of spacer layers. The green line shows the linear fitting (R^2 = 0.999).

"4) Eq. 5: I am confused that the cell length L enters the analysis. Would the patterns (onset of LP) be different if cells of say 1 vs 10 cm are used? Also ε^2Ra depends on L! Moreover, microgravity conditions would yield Ra=0, but I doubt that LPs would form in thick layers for g = 0."

Author reply: We thank the Reviewer for this valid issue. The Reviewer is right; the cell length does not affect the pattern formation in our study. We revised the calculations and updated the results. In the case of $g = 0$, the product $\varepsilon^2 Ra$ is zero, indicating Darcy flow in the system, generating the periodic precipitation in this condition.

Changes: We rewrote the corresponding part and updated Figure 5b and Table S1.
(i) Page 19.

$$Ra = \frac{g\Delta\rho_S {a^*}^2 b^*}{12\mu D}, \quad (5)$$

$$\varepsilon = \frac{a^*}{b^*\sqrt{12}}, \quad (6)$$

where g, a^*, b^*, μ, and D are the standard acceleration of gravity, the smallest length of the cell, the length of the cell parallel with the gravity acceleration vector, the dynamic viscosity of the medium, and the molecular diffusion coefficient, respectively. Also, $\Delta\rho_S$ is the density difference between solute-saturated fluids and solute-free fluids. In our setup, both a^* and b^*equal d and equations 5 and 6 can be rewritten in the form:

$$Ra = \frac{g\Delta\rho_s d^3}{12\mu D}, \quad (7)$$
$$\varepsilon = \frac{d}{d\sqrt{12}} = \frac{1}{\sqrt{12}}. \quad (8)$$

(ii) Pages 19 and 20.
In our investigation, we observed that the last appearance of the periodic structure with the increasing cell gap occurred at a value ($\varepsilon^2 Ra$ = 3.2 in the case of d = 120 μm), which is close to the value corresponding to the boundary between these two flow regimes ($\varepsilon^2 Ra$ = 1), and the LPs were observed at the regime of Hele-Shaw flow.

(iii) Figure 5

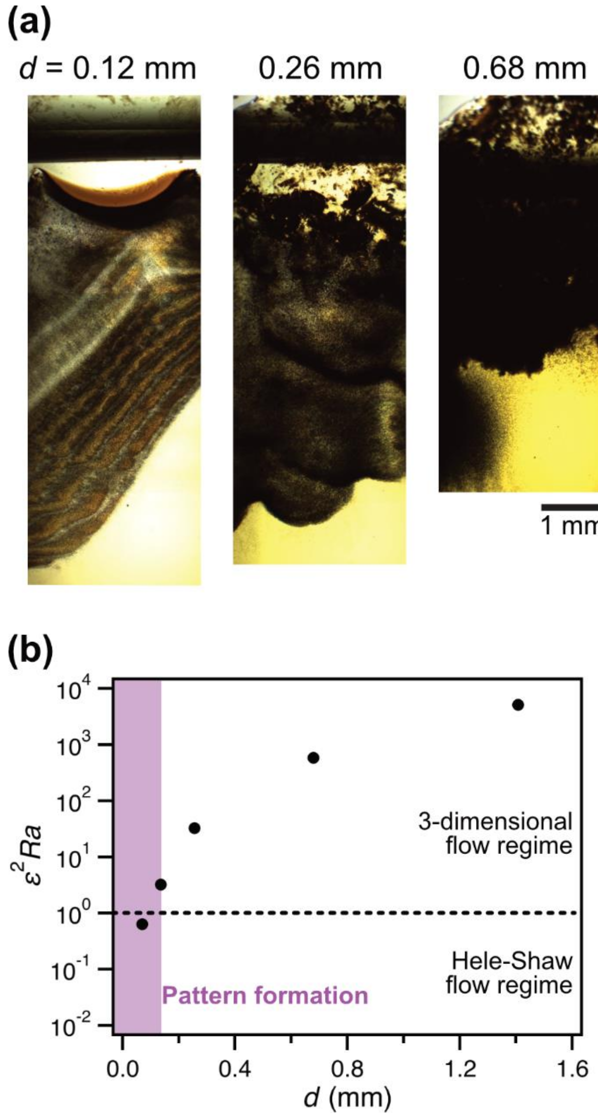

Figure 5. Effect of vertical gap distance (d) on the pattern formation of $CuCrO_4$ system in both the HS cell and a hand-crafted HS cell. (a) Pattern formation in the hand-crafted HS cells with different

d values for $[CuCl_2]_0$ = 1.0 M and $[K_2CrO_4]_0$ = 0.2 M: *d* = 0.12 mm, 0.26 mm, and 0.68 mm. (b) Variation of the Rayleigh–Darcy number with the cell anisotropy ratio ($\varepsilon^2 Ra$) as a function of *d*. The purple square indicates the region where the periodic pattern was formed. The dotted line corresponds to the 2D HS and 3D flow regimes boundary.[68,69] Error bars were obtained from 3 replicates calculated using p-values < 0.05.

(iv) Table S1 in the SI.

Table S1. Parameters and results for calculating the Rayleigh-Darcy number (*Ra*) and the geometry parameter (ε).

d (m)	g (m s^{-2})	$\Delta\rho_s$ (kg m^{-3})^a	D (m^2 s^{-1})^b	μ (Pa s)^c	Ra	$\varepsilon^2 Ra$
7.0×10^{-5}	9.8	27	1.0×10^{-9}	9.9×10^{-4}	7.6	6.4×10^{-1}
1.2×10^{-4}					3.9×10^{1}	3.2×10^{0}
2.6×10^{-4}					3.9×10^{2}	3.2×10^{1}
6.8×10^{-4}					7.0×10^{3}	5.8×10^{2}
1.4×10^{-3}					6.1×10^{4}	5.1×10^{3}

“5) While I am not requesting these data, it might be interesting to repeat the experiment in a wedge-shaped HS cell with heights between say 20 and 200 um.”

Author reply: We thank the Reviewer for this suggestion. Investigating periodic precipitation in a wedge-shaped HS cell could be the next step in exploring this phenomenon in a liquid layer, but this type of investigation is outside the scope of this study. Pattern formation in a wedge-shaped HS provides complexity due to the cell's shape. The height increase can influence the stability of the colloids formed in the liquid layer (due to the changing of the Rayleigh–Darcy number) and the diffusion flux of the chemical species.

Changes: Page 22. We added a short discussion to highlight this issue in the conclusion.
A more complex approach could be the investigation of the formation of periodic precipitation in a wedge-shaped HS. This setup affects not only the fluidity of the medium but the diffusion flux of the chemical species, contributing to the creation of more complex chemical structures in reaction–diffusion systems.

“6) In the Science paper https://doi.org/10.1126/science.aah6350, the Aizenberg group shows some indication of Liesegang patterns formed by biomorphs in microfluidic devices (see Fig. 1G, see also the SI). The results are not fully convincing, but might be worth mentioning (or at least of interest to the authors).”

Author reply: We thank the Reviewer for this issue. We found this paper interesting. Therefore, we added a short discussion in the text and cited the paper.

Changes: Page 5.
It should be noted that in the work of Nadir Kaplan *et al.*, the growth of coral-like structures produced three zones in a microfluidic chamber with a height of 150-200 μm.[51]

"7) Can the authors provide more information on the size of the crystals formed in their different systems. Is the crystal height comparable to the layer height? Should we expect differences between a full and partial blockage of diffusion by these crystals?"

Author reply: This is a valid point. Based on the Reviewer's suggestion, we carried out experiments in a Hele-Shaw cell with a thickness of 70 μm and analyzed the formed crystals using optical and scanning electron microscopies. We obtained that the average crystal size is a few μm, and based on this result, we can conclude that the formed crystal cannot block the diffusion of the reagents.

Changes:
(i) Page 13. We added a short discussion of the results in the text.
We performed optical and scanning electron microscopy (SEM) measurements to investigate the size of the formed particles. We obtained that the size of the particles was between 2 and 10 μm, indicating that they did not entirely block the diffusion of the reagents.

(ii) SI, Pages S7 and S8. We prepared new figures showing the crystals under optical and scanning electron microscopes.

Budapest University of Technology and Economics
Faculty of Natural Sciences • Institute of Physics
Fizika Tanszék

1111 Budapest, Budafoki út 8. F.épület, Hungary
Phone: +36 1463-2312, Fax: +36 1463 4180
http://dept.phy.bme.hu

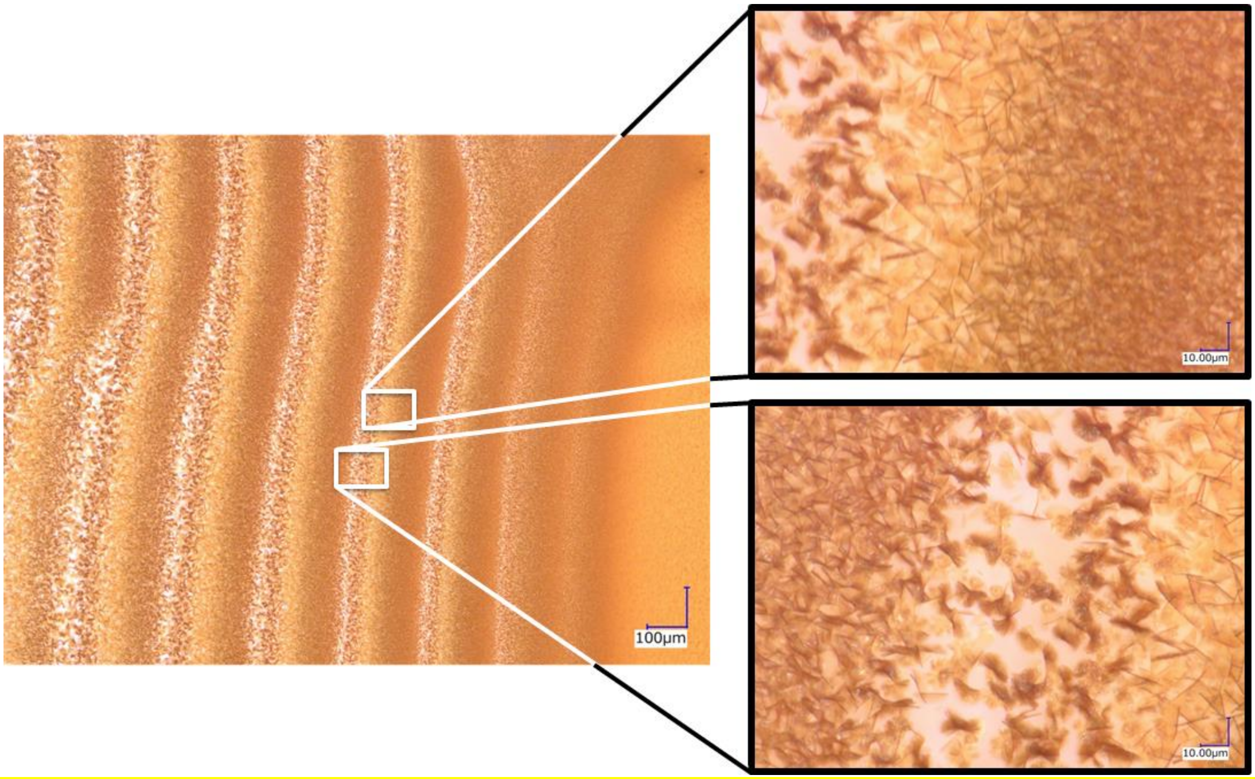

Figure S2. Optical micrographs of the pattern formed in HS cell with *d* = 70 μm under the following conditions: $[CuCl_2]_0$ = 1.0 M and $[K_2CrO_4]_0$ = 0.2 M.

Budapest University of Technology and Economics
Faculty of Natural Sciences ● Institute of Physics
Fizika Tanszék
1111 Budapest, Budafoki út 8. F.épület, Hungary
Phone: +36 1463-2312, Fax: +36 1463 4180
http://dept.phy.bme.hu

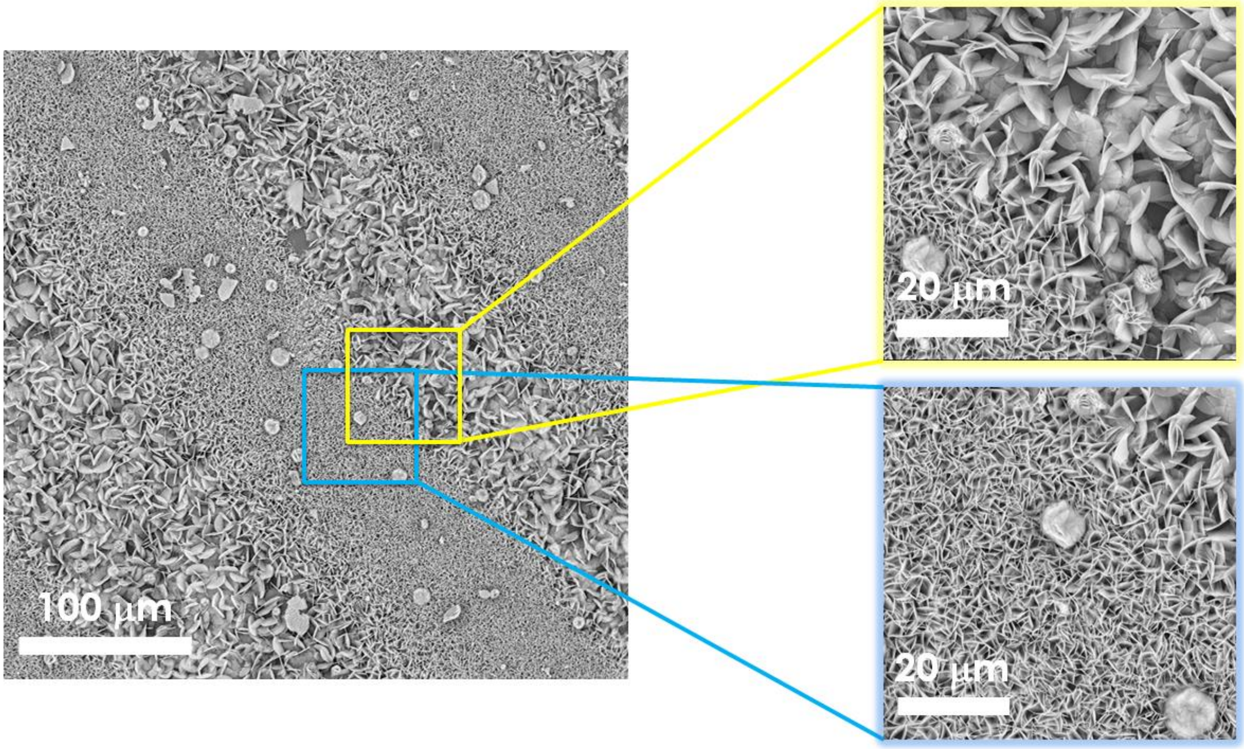

Figure S3. SEM micrographs of the pattern formed in the bottom glass slide of the HS cell with *d* = 70 µm under the following conditions: $[CuCl_2]_0$ = 1.0 M and $[K_2CrO_4]_0$ = 0.2 M.

(iii) SI, Page S3. We added a short description of the sample preparation and instrumentation.

1.3 Sample preparation for the scanning electron microscopy (SEM)

The solution of K_2CrO_4 (0.2 M, 5 µL) was sourced into the narrow space between the slide glass and cover glass, and then, $CuCl_2$ aqueous solution (1.0 M, 3 µL) was added. After the pattern formation, the reactant solutions were removed by blotting paper, and the cover and slide glasses were separated and dried at ambient conditions. Gold sputtering was performed to maintain appropriate electrical conductance. The morphology of precipitates was investigated using a scanning electron microscope (Phenom proX PREMIUM, Phenom World).

"8) Does the surface (what is the material?) of the HS cell affect crystal growth? Would different materials potentially create different outcomes or do the crystals nucleate in solution?"

Author reply: We thank the Reviewer for this comment. As described in the experimental section, the HS cell is made of glass. Due to the heterogeneous nucleation, different cell materials may affect pattern formation. This issue is essential and can be investigated in a separate study. However, based on the observations, nucleation and crystal growth occur in the solution.

“9) P. 21: rephrase “>empirical stereotypes< of experiments in use”.”

Author reply: We rephrased the corresponding term in the text.

Changes: Page 21.
By showing a breakthrough that challenges the classical experimental setups, in which only gels have been used as reaction media for over a century since the discovery of the phenomenon, …

“10) Check for missing subscripts (e.g. caption 5, SI text at the end of the main paper).”

Author reply: We checked for the missing subscripts.

Changes:
(i) Page 20.
Figure 5. Effect of vertical gap distance (*d*) on the pattern formation of $CuCrO_4$ system in both the HS cell and a hand-crafted HS cell. (a) Pattern formation in the hand-crafted HS cells with different *d* values for $[CuCl_2]_0$ = 1.0 M and $[K_2CrO_4]_0$ = 0.2 M: *d* = 0.12 mm, 0.26 mm, and 0.68 mm.

(ii) SI, Page S4
Video S1. Periodic precipitation pattern formation in a liquid phase in copper chromate systems ($[CuCl_2]_0$ = 1.0 M, $[K_2CrO_4]_0$ = 0.2 M, and *d* = 70 μm). The video plays for 125 seconds and has a domain size of 4.0 × 3.0 mm.

<u>Reviewer #2</u>

“This manuscript describes the periodic precipitation patterns using a Hele-Shaw cell in a confined liquid phase. I think that the authors have reported a really interesting phenomenon. In addition to the interesting phenomenon, the authors have also investigated the phenomenon in good amount of depth, both experimentally and theoretically. The conclusion arrived based on the investigations is thus strong. The demonstration of pattern formation in a gel-free environment is indeed interesting. I think this manuscript is very suitable for the Journal of Physical Chemistry Letters.”

Author reply: We thank the Reviewer for his/her comments and the positive assessment of our work.

Budapest University of Technology and Economics
Faculty of Natural Sciences ● Institute of Physics
Fizika Tanszék
1111 Budapest, Budafoki út 8. F.épület, Hungary
Phone: +36 1463-2312, Fax: +36 1463 4180
http://dept.phy.bme.hu

This completes our reply. We again thank the Referees for their comments/suggestions and critical reading of our work. We hope that the manuscript is now in an acceptable form.

Sincerely yours,

István Lagzi

Budapest University of Technology and Economics
Faculty of Natural Sciences • Institute of Physics
Fizika Tanszék

1111 Budapest, Budafoki út 8. F.épület, Hungary
Phone: +36 1463-2312, Fax: +36 1463 4180
http://dept.phy.bme.hu
